# Powering single-cell genomics to unravel circulating tumour cell subpopulations in non-small cell lung cancer patients

**DOI:** 10.1007/s00432-022-04202-y

**Published:** 2022-07-28

**Authors:** Emmanuel Acheampong, Michael Morici, Afaf Abed, Samantha Bowyer, Du-Bois Asante, Weitao Lin, Michael Millward, Elin S. Gray, Aaron B. Beasley

**Affiliations:** 1 School of Medical and Health Sciences, Edith Cowan University, Perth, Joondalup, WA 6027 Australia; 2 Centre for Precision Health, Edith Cowan University, Joondalup, WA 6027 Australia; 3 Department of Medical Oncology, Sir Charles Gairdner Hospital, Hospital Avenue, Nedlands, WA 6009 Australia; 4 Harry Perkins Institute of Medical Research, Nedlands, WA 6009 Australia; 5 School of Medicine and Pharmacology, University of Western Australia, Crawley, WA 6009 Australia; 6 Linear Clinical Research, Hospital Avenue, Nedlands, WA 6009 Australia

**Keywords:** Circulating tumour cells, Epithelial-to-mesenchymal transition, Vimentin, Single-cell sequencing, Non-small cell lung cancer

## Abstract

**Background:**

Circulating tumour cells (CTCs) are attractive “liquid biopsy” candidates that could provide insights into the different phenotypes of tumours present within a patient. The epithelial-to-mesenchymal transition (EMT) of CTCs is considered a critical step in tumour metastasis; however, it may confound traditional epithelial feature-based CTC isolation and detection. We applied single-cell copy number alteration (CNA) analysis for the identification of genomic alterations to confirm the neoplastic nature of circulating cells with only mesenchymal phenotypes.

**Methods:**

We isolated CTCs from blood samples collected from 46 NSCLC patients using the Parsortix system. Enriched cells were subjected to immunofluorescent staining for CTC identification using a multi-marker panel comprising both epithelial and mesenchymal markers. A subset of isolated CTCs was subjected to whole genome amplification (WGA) and low-pass whole-genome sequencing (LP-WGS) for the analysis of copy number alterations (CNAs).

**Results:**

CTCs were detected in 16/46 (34.8%) patients, inclusive of CK^+^/EpCAM^+^ CTCs (3/46, 6.5%) and Vim^+^ CTCs (13/46, 28.3%). Clusters of Vim^+^ cells were detected in 8 samples, which constitutes 50% of the total number of NSCLC patients with CTCs. No patients had detectable hybrid CK^+^/EpCAM^+^/Vim^+^ cells. All of the tested CK^+^/EpCAM^+^ CTCs and 7/8 Vim^+^ CTCs or CTC clusters carried CNAs confirming their neoplastic nature. Notably, the Vim^+^ cluster with no CNAs was characterised by spindle morphology and, therefore, defined as normal mesenchymal circulating cells.

**Conclusion:**

Our results revealed that CK-negative, vimentin-expressing cells represent a large proportion of CTCs detected in NSCLC patients, which are likely missed by standard epithelial-marker-dependent CTC categorisation.

**Supplementary Information:**

The online version contains supplementary material available at 10.1007/s00432-022-04202-y.

## Background

Lung cancer is the worldwide leading cause of cancer mortality, with an estimated number of deaths reaching 1.8 million in 2020 alone (Sung et al. 2021). Non-small cell lung cancer (NSCLC) accounts for 80–85% of lung cancer cases (Zappa and Mousa 2016). NSCLC patients are typically diagnosed at an advanced stage, and the estimated 5-year survival rate for NSCLC is 26.4% (Ganti et al. 2021). Immunotherapy has revolutionised the treatment paradigm of NSCLC, significantly prolonging the overall survival of advanced-stage patients (Chiang and Herbst 2020; Berghmans et al. 2020). Despite the striking clinical improvement with immunotherapies, the majority of patients eventually fail to respond to these drugs due to the evolution of primary or secondary resistance (Borghaei et al. 2015; Brahmer et al. 2012).

Unravelling aggressive tumour cell phenotypes as they evolve during treatment can provide predictive insights into the occurrence of resistance to standard of care treatments. Circulating tumour cells (CTCs) have emerged as a minimally invasive “liquid biopsy” strategy that has particular relevance for NSCLC due to the complexities of obtaining a lung biopsy (Alix-Panabières and Pantel 2016; Keller and Pantel 2019). CTCs are tumour-derived cells shed from diverse neoplastic deposits in the bloodstream (Keller and Pantel 2019; Alix‐Panabières and Pantel 2017). As such, CTCs mirror tumour heterogeneity of both primary tumours and their metastases, making them excellent candidates that reflect the phenotypes of all lesions present within a patient at any one time (Hanssen et al. 2015). Moreover, while it is difficult in clinical practice to obtain repeat biopsies from any lesion, CTCs acquired through a sample of blood could be used as a source of information on the tumour tissue over time (Lucci et al. 2012).

Historically, analyses of CTCs mainly focused on cells expressing epithelial phenotype-specific markers, such as pan-cytokeratin (pCK) and epithelial adhesion molecule (EpCAM) for immunofluorescent identification (Cote and Datar 2016; Maly et al. 2019). Classical NSCLC CTCs are defined by an intact nucleus, expression of pCK and EpCAM which show the epithelial origin of the cells, and absence of CD45, indicating the cell is not of a hematopoietic lineage (Krebs et al. 2012). However, the epithelial-to-mesenchymal transition (EMT) is a key process for cells to leave the tumour and migrate into circulation. During this process, epithelial makers are down-regulated concomitantly with the upregulation of mesenchymal markers such as vimentin and N-cadherin (Tania et al. 2014; Bartis et al. 2014). Hence, mesenchymal cells may be missed when CTC isolation and identification are based only on epithelial features (Xu et al. 2015). This is particularly relevant for NSCLC, given that some studies have reported the presence of CTCs with mesenchymal features in patients affected by this malignancy (Li et al. 2020; Lecharpentier et al. 2011; Ciccioli et al. 2021). To overcome this challenge, we employed a novel epitope-independent isolation method using the Parsortix, a microfluidic device which captures CTCs based on the larger size and less deformable nature of tumour cells compared to normal blood cells (Chudziak et al. 2016).

By combining microfluidic enrichment for NSCLC CTCs together with immunofluorescence, we identified circulating cells with mesenchymal features in NSCLC patient samples. However, studies have not provided direct proof of the neoplastic nature of such mesenchymal cell subpopulations.

We applied single-cell copy number alteration (CNA) analysis for the identification of genomic alterations to confirm the neoplastic nature of these circulating cells with mesenchymal phenotypes.

## Materials and methods

### Patient recruitment and sample collection

A total of 46 NSCLC patients at Sir Charles Gairdner Hospital (SCGH) and Fiona Stanley Hospital (FSH) in Perth, Western Australia, were prospectively recruited between August 2018 and October 2021 before commencing treatment. Written informed consent was obtained from all patients and procedures were approved by Human Research Ethics Committees at Edith Cowan University (No. 18957) and Sir Charles Gairdner Hospital (No. 2013-246, RGS0000003289) in compliance with the Declaration of Helsinki. Experiments were conducted per institutional and national guidelines and regulations. For CTC quantification, at least 8 mL of blood from each subject was collected in vacutainer K_2_EDTA tubes (BD Biosciences, East Rutherford, NJ), following the collection of a serum tube to remove any potential contaminating epithelial or endothelial cells. Samples were processed within 6 h of blood collection.

### CTC isolation and identification

CTCs were enriched using the Parsortix system (Angle plc, Guildford, UK). Enriched cells were harvested according to the manufacturer’s instructions and fixed for 10 min at room temperature (RT) with 2% PFA. Following this, enriched cells were cytospun using Cytospin 4 (Thermo Fisher Scientific) onto glass slides at 2000 rpm for 5 min. For CTC identification, enriched cells were immunofluorescently stained based on a protocol developed in our lab (Spencer 2020). Fixed samples were fluorescently labelled with Alexa Fluor (AF) 488 and FITC-conjugated antibodies against epithelial markers (pan-cytokeratins and EpCAM), phycoerythrin (PE)-conjugated white blood cell (WBC) markers (CD45 and CD16) to exclude hematopoietic cells, an anti-PD-L1 antibody (28.8-AF647) for PD-L1 expression and 4′,6-diamidino-2-phenylindole (DAPI) for nuclear staining (Table S1). CTCs were defined as CK^+^/EpCAM^+^/CD45^−^/16^−^ with a DAPI^+^ nucleus. For mesenchymal marker expression analysis, previously stained slides underwent fluorescence quenching with an established protocol using sodium borohydride (Adams et al. 2016; Manjunath et al. 2019; Spencer 2020). This protocol allows multi-phenotypic subtyping of CTCs utilising sequential fluorescent quenching and re-staining for further biomarkers (Adams et al. 2016; Acheampong et al. 2022). Following quenching of fluorescence from the initial round of immunostaining, samples were re-stained with mesenchymal markers (vimentin-AF647, and N-cadherin-PE). Slides were visualised and scanned using an Eclipse Ti-E inverted fluorescent microscope (Nikon, Chiyoda, Japan). Images were analysed using the NIS-Elements Analysis software, version 5.21 (Nikon).

### Characterisation of enriched cells at the single-cell level

To confirm the malignant nature of the putative CTC populations by detecting genome-wide CNAs, the CellCelector (ALS, Jena, Germany) platform was employed to pick individual pCK and vimentin (Vim) only expressing cells from stained slides, previously optimised in our lab (Beasley et al. 2018). Picked cells were subjected to whole-genome amplification (WGA) using the Ampli1 WGA Kit (Silicon Biosystems, Bologna, Italy) according to the manufacturer’s specifications. Quality control of WGA-DNA was performed using Ampli1 QC Kit following the manufacturer’s instructions (Silicon Biosystems). WGA-DNA was used to construct 400 bp sequencing libraries using the Ampli1 LowPass Kit for Ion Torrent (Silicon Biosystems) following the manufacturer’s instructions. Pooled library was diluted to 50 pM and loaded into an Ion 530 Chip (Life Technologies) using the Ion Chef (400 base chemistry) (Life Technologies) and sequenced on an Ion S5 (Life Technologies) for 525 flows. CNAs were analysed using the Ion Reporter Software (Life Technologies).

### Statistical analysis

Demographic data were presented as numbers, ranges, counts, percentages, means, and medians. The graph for CTCs counts was processed using GraphPad Prism version 8.0.2. The primary endpoints of the outcome analysis were overall survival (OS) and progression-free survival (PFS). Kaplan–Meier method was used to estimate median OS and PFS within groups and differences in patient survival rates were determined using log-rank tests. Univariate Cox regression hazard model for OS was performed for age, sex, NSCLC stage, ECOG performance status, histological type, tumour PD-L1 expression, treatment, and CTC counts. All survival analyses were performed in R version 4.05 using the package “survplot”. *P* values less than 0.05 were considered statistically significant.

## Results

### Demographics of patients

The patient demographics are summarised in Table 1. The median age of patients was 72 years. Majorities of the patients were male (65.2%), had an Eastern Cooperative Oncology Group (ECOG) performance status of 0–1 (69.5%), and were smokers (95.6%). Most patients had stage IV M1c NSCLC (60.8%), and adenocarcinoma was the most histological type (78.3%) among patients. Fourteen patients had tumours with KRAS mutations (30.4%). Information on tumour PD-L1 expression was available for most patients, with most tumour biopsies (65.2%) expressing PD-L1 in 1% or more of tumour cells.

**Table 1:** Patient characteristics

Variables	*n* = 46	%
Age, years (median, IQR)	72 (64–76)	
Age group (years)		
< 70	18	39.1
≥ 70	28	60.9
Sex		
Male	30	65.2
Female	16	34.8
ECOG status		
0	19	41.2
1	13	28.3
2	13	28.3
Unknown	1	2.2
Smoking status		
Smoker	44	95.6
Non-smokers	1	2.2
Unknown	1	2.2
Histological type		
Adenocarcinoma	36	78.3
Squamous cell carcinoma	8	17.4
Others	2	4.5
Stage		
IIIB	1	2.2
M1a	12	26.1
M1b	5	10.9
M1c	28	60.8
Molecular status		
KRAS mutant	14	30.4
No detection	20	43.5
Unknown	12	26.1
Treatment		
IO	16	34.8
Chemotherapy + IO	27	58.6
None	3	6.5
Tumour PD-L1 expression (%)		
< 1	11	2.9
1–49	13	28.3
≥ 50	17	36.9
Undetermined^a^	5	10.9

*ECOG* Eastern Cooperative Group, *IO* immunotherapy, *IQR* inter-quartile range

^a^Undetermined due to insufficient tumour material

### Enrichment and detection of CTCs

**Fig. 1:**
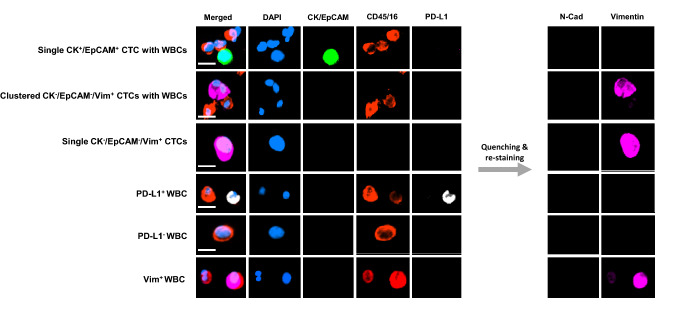
Representative fluorescence images of cells enriched with the Parsortix system. Cells were stained with antibodies for pan-cytokeratins and EpCAM (green), CD45 and CD16 (red), and PD-L1 (white) to identify and characterise classical NSCLC CTCs, followed by fluorescence quenching and re-staining with antibodies for N-cadherin (cyan) and vimentin (pink). Scale bar represents 10 μm

All enriched samples had two rounds of immunofluorescence staining for CTC characterisation. CTCs were initially identified as CK^+^/EpCAM^+^, DAPI^+^, CD45^−^/16^−^, and with or without PD-L1 expression. The fluorophores were quenched using borohydride and the samples were re-stained with vimentin and N-Cadherin. Different CK^+^/EpCAM^+^ and Vim^+^ expression patterns observed in CTCs from patients are shown in Fig. 1.

**Fig. 2:**
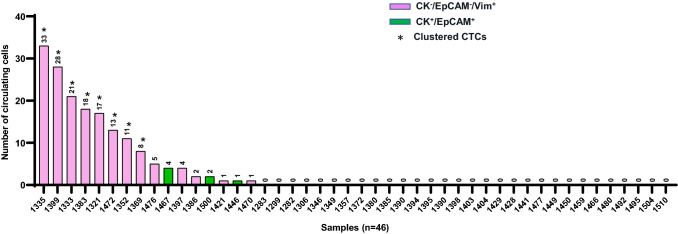
CTC counts in NSCLC patients. Blood samples collected before commencing treatment were enriched for CTCs using the Parsortix system (*n* = 46). The number of cells on each sample is indicated on top of the bars. Bar colours indicate whether identified CTCs expressed CK/EpCAM—green, or vimentin—purple. *Samples with CTC clusters

In the 46 NSCLC patients, CK^+^/EpCAM^+^ cell types were found in 3/46 (6.5%) patients’ samples (range 1–4 cells/sample) (Fig. 2). Surprisingly, none of the CTCs were positive for PD-L1 expression. However, all patients had a proportion of their WBCs expressing PD-L1 (Figure S1). Cells expressing only vimentin (CK^−^/EpCAM^−^/Vim^+^) with negative for CD45/16 expression were found in 13/46 (28.3%) samples (range 1–33 cells/sample). None of the patient samples had detectable hybrid CK^+^/EpCAM^+^/Vim^+^ CTCs. Of the 16 patients with detectable CTCs, 50% had CTC clusters all of which were vimentin-positive only. The number of cells within clusters ranged from 2 to 10 cells. All the CK^+^/EpCAM^+^ cells were present as single cells. None of the analysed patients’ samples had both CK^+^/EpCAM^+^ and Vim^+^ cell types. Overall, a total of 169 CTCs (range 1–33) were detected in 16/46 (34.8%) patients’ samples (Fig. 2).

### Copy number alteration analysis of detected circulating cells from NSCLC patients

**Fig. 3:**
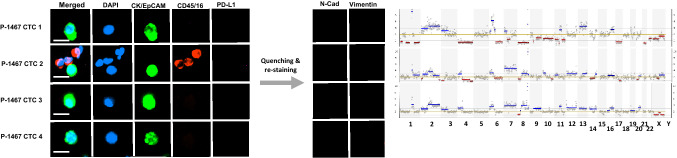
CNA profiles of epithelial CTCs. Immunostaining and morphology of CK^+^/EpCAM^+^ CTCs in relation to their CNA profiles obtained from low-pass whole-genome sequencing. Blue lines indicate copy gains and red lines copy number losses

**Fig. 4:**
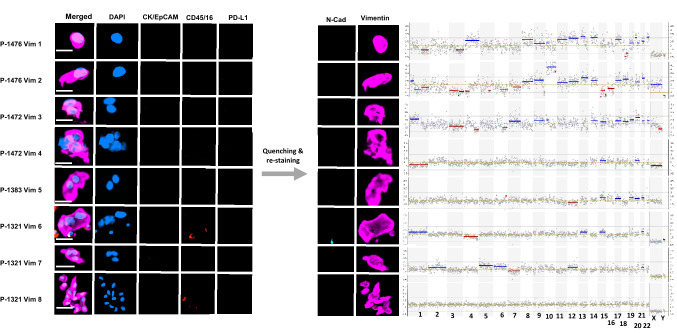
CNA profiles of mesenchymal CTCs. Immunostaining and morphology of vimentin-expressing CTCs in relation to their CNA profiles obtained from low-pass whole-genome sequencing. Blue lines indicate copy gains and red lines copy number losses

To confirm the malignancy of the Vim^+^ putative CTCs found as clusters or single cells, we utilised WGA and LP-WGS to detect chromosomal CNAs. Additionally, EpCAM^+^/CK^+^ CTCs found in patients 1467 and 1500 were also analysed. Overall, we obtained genomic profiles from a total of 13 CTCs or CTC clusters (Figs. 3, 4). In addition, four WBCs were also analysed as negative controls of any potential artifacts during the WGA (Figure S2). Patient 1467 had four classical CK^+^/EpCAM^+^ CTCs detected, which were picked and analysed for CNA (Fig. 3). Three of the CTCs displayed multiple CNAs and similar profiles despite some heterogeneity between cells. One of the four CTCs had low-quality sequencing and CNAs were not assessable (Fig. 3, CTC 4). The morphology of this cell suggests that it is undergoing apoptosis or has been damaged.

We also analysed the genomic profiles of single and clusters of putative CTCs identified via vimentin expression (Fig. 4). These Vim^+^ cells displayed heterogeneous chromosomal CNA distribution patterns. The two single cells isolated from 1476 displayed similar CNA profiles, while the two clusters from 1472 were distinct. Only one cluster of two cells was analysed from 1383, and it was found to have CNAs. Finally, three clusters of Vim + cells were obtained from 1321; two of the clusters showed CNAs while the third displayed a ‘flat’ normal diploid CNA profile comparable to WBC controls (Figure S2). Notably, the third cluster of Vim + cells displayed a distinct spindle morphology (Fig. 4, Vim 8).

### Survival analysis

**Fig. 5:**
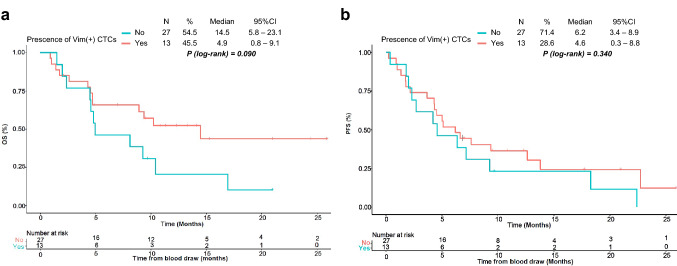
Kaplan–Meier curves for OS (**a**) and PFS (**b**) of patients with and without vimentin-expressing–Vim( +) CTCs. Log-rank *p* values, group numbers, percentages, median, and 95% CIs are indicated for each plot

We analysed the association of patient characteristics and CTC counts with clinical outcomes, OS, and PFS in participants with a minimum of 6 months of follow-up time and that were treated with systemic therapies (*n* = 40). A threshold of one Vim^+^CTC was used for survival analysis because of the low number of detected CTCs. The median follow-up for OS and PFS for the cohort were 12.3 months (95% CI 9.5–20.9) and 12.2 months (95% CI: 9.4–17.7), respectively. Log-rank survival analysis showed similar median PFS for patients with ≥ 1 Vim^+^ CTCs compared to patients without Vim^+^ CTCs. However, patients with ≥ 1 Vim^+^ CTCs had a median OS of 4.9 months compared to 14.5 months observed for those with no Vim^+^ CTCs (Fig. 5).

Cox regression models again showed no difference in PFS (HR = 1.42, 95% CI 0.68 to − 2.94, *p* = 0.341), while Vim^+^ CTCs was associated with worse OS (HR = 1.98, 95% CI 0.89–4.45, *p* = 0.096), albeit not statistically significant (Fig. 5 and Table 2). There were no statistically significant associations of the patients' demographics with clinical outcomes, with regard to age, sex, ECOG status, stage of disease, tumour PD-L1 expression, and histological type (Table 2).

**Table 2:** Association of patients’ characteristics with clinical outcomes of treatment-naïve NSCLC patients

Variables	OS		PFS	
	HR (95% CI)	*P* value	HR (95% CI)	*P* value
Sex—female vs. male	0.87 (0.39–1.98)	0.756	0.75 (0.36–1.57)	0.449
Age (years)—< 70 vs. ≥ 70	1.00 (0.45–2.25)	0.995	1.51 (0.71–3.20)	0.282
ECOG performance status—0 vs. ≥ 1	1.66 (0.72–3.82)	0.232	1.79 (0.83–3.87)	0.135
Histological type—SCC vs. ADC	1.01 (0.39–2.58)	0.985	0.82 (0.38–1.76)	0.610
Stage—M1a/b vs. M1c	1.56 (0.64–3.29)	0.366	1.31 (0.64–2.68)	0.463
Treatment—IO vs. Chemo + IO	1.40 (0.58–3.39)	0.458	1.10 (0.53–2.31)	0.792
Tumour PD-L1 expression (%)—0 vs. ≥ 1	0.91 (0.37–2.21)	0.838	0.84 (0.38–1.85)	0.669
Vim^+^CTC—No vs. Yes	1.98 (0.89–4.45)	0.096	1.42 (0.68–2.94)	0.341

*P* < 0.05 indicates statistical significance

*ECOG* Eastern Cooperative Group, *SCC* squamous cell carcinoma, *ADC* adenocarcinoma, *OS* overall survival, *PFS* progression-free survival, *HR* hazard ratio, *CI* confidence interval

## Discussion

Currently, the EpCAM/CK-dependent CellSearch system remains the only FDA-approved CTC enumeration platform. However, this system fails to recognise CTCs that have downregulated epithelial markers while undergoing EMT. This leads to an underestimation of the CTCs undergoing EMT with hybrid or mesenchymal phenotypes (Yin et al. 2015; Yu et al. 2015).

Our study employed the Parsortix system for CTC enrichment and an immunofluorescence protocol that combines both epithelial and mesenchymal markers for CTC identification. Our detection rate (34.8%) was considerably lower than those obtained in most of the previous studies that used the Parsortix system. Janning et al. reported a detection rate of 61% (Janning et al. 2019), and Papadaki et al., detected CTCs in 60% of total patients (Papadaki et al. 2020). However, the observed detection in this study was similar in comparison to a study by Mondelo-Macia et al., who reported a CTC detection rate of 35% in metastatic NSCLC patients using the Parsortix (Mondelo‐Macía et al. 2021). These previous studies used CK and EpCAM expression as positive markers and CD45 expression as the only negative WBC marker for CTCs. This restricted definition of CTCs does not take into account the variation in WBCs in the bloodstream with little or absent CD45 expression such as neutrophils (Gorczyca et al. 2011). Compounding the issue to a higher extent is the evidence that neutrophils stain positive for CK (Schehr et al. 2016; Streicher et al. 1998), raising concerns about the specificity of the traditional definition of CTCs. Beyond CD45, this study used CD16 for WBC identification, which has been found to significantly reduce the number of false positive CTCs (Swennenhuis et al. 2016; Spencer 2020). These factors may explain the low CTC detection rate in our study when compared to others.

One unanticipated finding was that in our cohort none of the detected NSCLC CTCs had PD-L1 expression, despite 65.2% of the cohort having PD-L1-positive tumours (> 1%), with 36.5% with more than 50% tumour specific staining. Published studies have consistently reported wide ranges of the rate of PD-L1 expressing CTCs, from 8 to 100%, in NSCLC (Acheampong et al. 2020; Kong et al. 2021; Ouyang et al. 2021). Detection of PD-L1 expression is influenced by a range of different factors including different antibodies, cut-off values, and CTC isolation platforms (Ouyang et al. 2021; Acheampong et al. 2020). Nevertheless, we detected PD-L1 expression on a subset of WBCs present in the enriched samples which is consistent with reports from previous studies (Ilié et al. 2018; Kotsakis et al. 2019).

EpCAM and different members of the cytokeratin family are frequently utilised for CTC identification before subsequent characterisation due to the epithelial nature of NSCLC (Hamilton and Rath 2016). Interestingly, the majority of the detected circulating cells in this study expressed vimentin with total loss of EpCAM and cytokeratin. More precisely, many of these Vim^+^ cells had cytomorphological characteristics such as shape and size consistent with CTCs, in addition to being negative for WBC markers (Boffa et al. 2017). Moreover, all putative NSCLC CTCs in this study were negative for N-cadherin. Since these identified circulating cells do not meet the field consensus criteria for CTCs, due to their lack of CK we assessed the neoplastic origin of these Vim^+^ cells together with CK^+^/EpCAM^+^ CTCs by LP-WGS analysis.

Classical CTCs presented with significant genomic alterations with some degree of heterogeneity. Among the Vim^+^ cells, those presenting CNAs could be considered bona fide CTCs. Our findings are in line with a previous study by Xu et al. who identified Vim^+^ circulating cells in metastatic prostate cancer patients using the Parsortix and confirmed their malignancy by genomic alterations (Xu et al. 2017). Another study by Reduzzi et al. demonstrated that enriched circulating cells lacking both epithelial and leukocyte marker expression presented altered CNA profiles and thus were defined as CTCs (Reduzzi et al. 2020). Because the authors only identified double negative cells for the CNA analysis, the actual phenotype of these cells was unknown. By contrast, our putative CTCs were phenotypically identified to be Vim^+^/CK^−^ cells (Reduzzi et al. 2020). These reports together indicate the relevance of the existence of CTCs subpopulations that express low or no EpCAM or CK and can escape detection by epithelial isolation methods and markers.

It is also worth noting that some Vim^+^ cells did not carry CNA and were, therefore, classified as non-tumourigenic mesenchymal-derived circulating cells. Vimentin is also expressed in circulating endothelial cells, which have been shown to also increase in cancer patients (Chen et al. 2021; Lin et al. 2017). The presence of hematopoietic cells of mesenchymal origin and reactive stromal cells compromise the specificity for recognition of CTCs via mesenchymal markers (Stoecklein et al. 2016; Schehr et al. 2016; Cima et al. 2016; Plaks et al. 2013). Preferably, additional exclusion markers should be included in future panels. Cancer patients have been shown to have an increased number of circulating endothelial cells (CECs) that are probably shed from tumour angiogenesis-associated processes or damaged tumour vessel walls (Ilie et al. 2014). Therefore, immunoassays for CTCs should include markers such as CD31, CD34, and CD144 to exclude cells of endothelial origin (Magbanua et al. 2015; Bidard et al. 2010).

The prognostic value of vimentin expression in various cancers has been well documented in the literature (Santamaria et al. 2017; Dongre and Weinberg 2019). High levels of vimentin expression are associated with poor survival in patients with NSCLC (Ye et al. 2016). Although our survival analyses in patients with Vim^+^ CTCs did not achieve statistically significant levels, they showed a trend towards unfavourable OS. A previous study by Zhang et al. indicated mesenchymal CTCs identified NSCLC patients with distant metastasis (Zhang et al. 2019).

Our study employed a robust CTC isolation, identification, and downstream single-cell genomic analysis. Nonetheless, some limitations need to be mentioned. The sample size of the study was small. We were not able to compare the CNA profiles of NSCLC CTCs to that of the matching tumours as samples were not available for profiling. Moreover, some Vim^+^ cells collected could not be used for CNA analysis due to unsuccessful amplification. This may have been caused by the method used to fix and immunostained the cells, which may have compromised the quality of the nucleus, or our inability to effectively recover the nuclei using the micromanipulator (CellCelector).

## Conclusion

Overall, these results reveal that pCK negative, EpCAM negative, and vimentin expressing cells represent a large proportion of CTCs detected in NSCLC patients. These cells would be commonly missed by standard CTC categorisation. The presence of CNAs confirmed the neoplastic nature of a proportion of vimentin-only expressing cells. The lack of CNA in some of the CK^−^ Vim^+^ cells underscores the need for better markers to identify and quantify CTCs. Future longitudinal studies with larger cohorts are needed to validate the clinical validity of epitope-independent microfluidic technologies for CTC isolation and the consideration of the addition of Vim^+^ CTCs to the classical CTC definition in NSCLC and evaluation of their prognostic value.

## Supplementary Information

Below is the link to the electronic supplementary material.

Supplementary file1 (DOCX 371 KB)

## Data Availability

Raw data are available upon request.
