# Supplementary material for: Powering single-cell genomics to unravel circulating tumour cell subpopulations in non-small cell lung cancer patients
Source: J Cancer Res Clin Oncol. 2022 Jul 28;149(5):1941–50. doi: 10.1007/s00432-022-04202-y (PMC10097753; doi:10.1007/s00432-022-04202-y)
Supplement: Supplementary file 1 — Supplementary file1 (DOCX 371 KB) [file 432_2022_4202_MOESM1_ESM.docx]

**Supplementary files**

**Powering single-cell genomics to unravel circulating tumour cells subpopulations in non-small cell lung cancer patients**

Emmanuel Acheampong^1,2^, Michael Morici^1,2^, Afaf Abed^1,2,7^, Samantha Bowyer^3,6,7^, Du-Bois Asante^1,2^, Weitao Lin^1,2,5^, Michael Millward^1,2,6,7^, Elin S Gray^1,2*^, Aaron B Beasley^1,2^

**Table S1: Antibodies used for immunocytochemistry staining**

| Antibody | Host Species | Conjugate | Clone | Antigen location | Supplier (CTLG #) | Dilution | Use |
| --- | --- | --- | --- | --- | --- | --- | --- |
| CD16 | Mouse | PE | 3G8 | Membrane | BioLegend,San Diego USA, (302008) | 1/50 | WBC identification |
| CD45 | Mouse | PE | HI30 | Membrane | BioLegend, San Diego, USA (982310) | 1/50 | WBC identification |
| Cytokeratins | Mouse | FITC | CK3-6H5 | Cytoskeleton | Miltenyi Biotech, Gladbach, Germany (130-118-964) | 1/50 | CTC identification |
| Cytokeratins | Mouse | AF488 | C11 | Cytoskeleton | Cell Signalling Technology, Denver, USA (4523S) | 1/100 | CTC identification |
| Cytokeratins | Mouse | AF488 | AE1/AE3 | Cytoskeleton | ThermoFisher Scientific, USA, (53-9003-82) | 1/200 | CTC identification |
| EpCAM | Mouse | FITC | VU-1D9 | Membrane | ThermoFisher Scientific, USA, (A15755) | 1/100 | CTC identification |
| Vimentin | Mouse | AF647 | V9 | Cytoskeleton | Abcam, USA, (ab195878) | 1/1000 | CTC identification |
| N-Cadherin | Mouse | PE | 8C11 | Membrane | BioLegend USA, (350806) | 1/20 | N-cadherin expression |
| PD-L1 | Rabbit | AF647 | 28-8 | Membrane | Abcam, USA, (ab205921) | 1/400 | PD-L1 expression |

**PE:** **phycoerythrin, AF: Alexa Fluor, FITC: Fluorescein isothiocyanate**


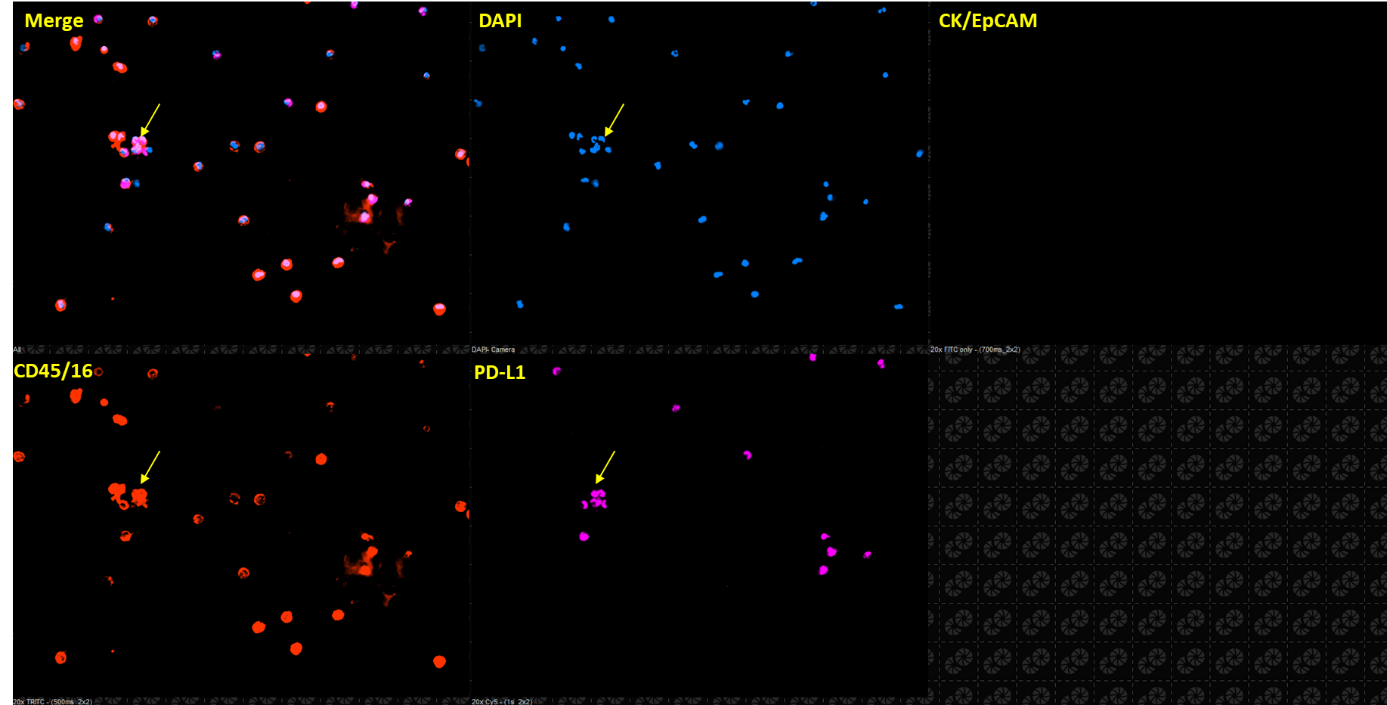


**Figure S1: Representative immunofluorescence images of Parsortix-enriched WBC expressing PD-L1.** (CK/EpCAM^-^, CD45/16^+^, PD-L1^+^). Cells were stained with DAPI (blue), anti-pCK/EpCAM (green), anti-CD45/16 (red) and anti-PD-L1 antibodies (pink). WBC cells are indicated with yellow arrows. Images were taken using an Eclipse Ti-E inverted fluorescent microscope (magnification X20).


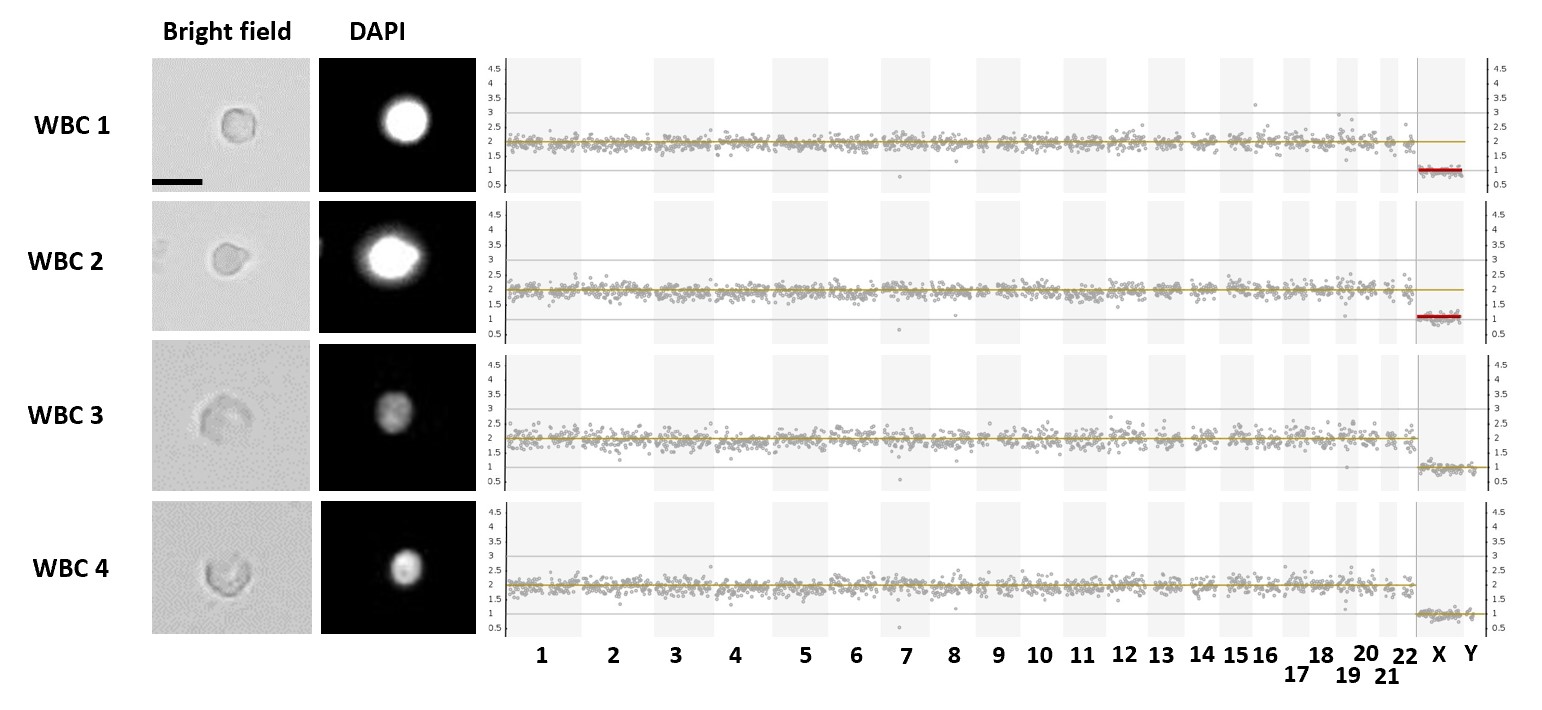


**Figure S2: CNA profiles of WBCs used as negative controls. Bright field and DAPI images of WBCs in relation to their genomic profiles obtained from low-pass whole-genome sequencing.**
